# Recent Medico-Legal Decisions

**Published:** 1889-10

**Authors:** 


### Selections.

### RECENT MEDICO-LEGAL DECISIONS.

The following abstracts of recent decisions in medico-legal cases are taken from the paper of Marshall D. Ewell, M. D., Attorney-at-law, published in the *North American Practitioner*:

### DRUGGISTS.

A graduate of a "school of pharmacy" which is one of the departments of an advanced institution of learning, such as the University of Michigan, whether situated in Kentucky or not, is a graduate of a "college of pharmacy," within the meaning of the Pharmacy Act. *State Pharmacy Board v. White*, 84 Ky., 626.

Graduation as a pharmaceutical chemist confers the honor of a "graduate in pharmacy." *Id.*

Under the Pharmacy Act of Kentucky, all "graduates in pharmacy" are entitled to be entered as registered pharmacists without submitting to an examination and without having served an apprenticeship, and any rule of the State Board of Pharmacy to the contrary is void. *Id.*

Mandamus will lie to compel the State Board of Pharmacy to enter a "graduate in pharmacy" as a registered pharmacist, since the board has no discretion in the matter. *Id.*

### DRUNKENNESS.

Voluntary drunkenness which precludes a comprehension of the nature of the act, or recognition of the person killed, is no excuse for murder; but *mania a potu*, or any insanity or permanent unsoundness of mind resulting from the use of intoxicating liquors, will exempt one who commits a murder from punishment therefor. *Beck v. State*, 76 Ga., 452.

### EMPLOYMENT.

A physician employed by the conductor of a train to care for a man injured by the train, can recover against the railroad company for his services if, after knowledge of his employment by the conductor, the company failed to notify him that it would not be responsible. *Terre Haute & I. R. Co. v. Stockwell*, (Ind.) 20 N. East, 650.

### EXPERT.

When a medical expert is asked to give his professional opinion to a jury, not upon matters within his own knowledge, but upon a hypothetical case founded upon the testimony of witnesses previously examined in the case, the questions to him must be so shaped as to give him no occasion to mentally draw his conclusion from the whole evidence, or a part thereof, and from these conclusions, so drawn, express his opinion, or to decide as to the weight of the evidence or credibility of the witnesses; and his answers must be such as not to involve any such conclusion so drawn, or any opinion of the expert as to the weight of the evidence or the creditability of witnesses. *Kerr v. Lunsford* (W. Va., 2 L. R. A. 668, 8 S. E., 493.

The opinion of medical experts, founded on testimony already in the case, can only be given on a hypothetical case; and the hypothesis must be clearly stated, so that the jury may know with certainty upon precisely what state of assumed facts the expert bases his opinion. *Id.*

In putting hypothetical questions to expert witnesses, counsel may assume the facts in accordance with their theory of them. It is not essential that he state the facts as they exist, but the hypothesis should be based on a state of facts which the evidence in the cause tends to prove. *Id.*

The opinion of an expert witness as to the nature and extent of an injury to a person is not inadmissible because based in part on the statements of the injured person. *Louisville, N. A. & C. R. Co. v. Snyder* (Ind.) 20 N. East, 284.

HEALTH, BOARDS OF.

A resolution of the board of health, authorizing a person to compel those having scarlet fever to remain at home, and provide for their wants as long as necessary for the public safety, gives him authority to provide a physician for them. *Wilkinson v. Long Rapids Twp.* (Mich.), 41 N. W., 861.

An action by a physician employed under authority of the board of health of a township, to recover for his services, is properly brought against the township. *Id.*

INSANITY.

Insanity is a fact that cannot be proven by reputation, or by a witness who is not an expert, unless he first gives the facts upon which his opinion is based. *Grubb v. State* (Ind.), 20 N. East, 257.

Where there is an issue made as to sanity, and evidence is introduced under it tending to show insanity, there is no presumption to be indulged one way or the other. *Missouri Pac. R. Co. v. Brazil* (Tex.), 10 S. W., 403.

A defendant in a criminal case who raises the defense of insanity must prove it by a preponderance of evidence; and this applies as well to the causal connection between the fact of insanity and the crime committed as to the insanity itself. *Gunter v. State*, 83 Ala., 96.

A person, though of weak mind, but with sufficient capacity to distinguish right from wrong in respect to the particular acts charged, is accountable for his acts, and the plea of insanity will be unavailing as a defense for crime. *Anderson v. State* (Neb.), 41 N. W., 357.

An instruction reads as follows: "If you believe from the evidence that defendant fired the shot that caused the death of the deceased, and that, at the time of the controversy, defendant was in such a mental condition as to distinguish the difference between right and wrong, then he was responsible for his act, and you must convict," is erroneous, as it does not, standing alone, state a correct legal proposition. *Kearney v. People*, 11 Colo, 258.

Moral insanity, as distinguished from mental derangement, is not an excuse for crime, and does not exempt from pnnishment therefor. *People v. Kerrigan*, 73 Cal., 222.

MEDICAL PRACTICE ACTS.

A State statute making it a misdemeanor, punishable by fine or imprisonment, to practice medicine without a certificate from the State board of health that the practitioner is a graduate of a reputable medical college, is not unconstitutional as depriving him of life, liberty or property, without due process of the law. *Dent v. West Virginia*, 129 U. S., 114; 32 L. ed., 623; 39 Alb. L. J., 189; 28 Cent. L. J., 262; 9 Sup. Ct. Rep., 231.

The words "suitable graduate in medicine," in the California Act providing for the appointment of a county physician, include one legally licensed to practice medicine and surgery under the laws of the State, although he was never graduated at any college, school or university which confers the degree of doctor of medicine. *People, Attorney-General v. Eichelrath* (Cal.), 2 L. R. A. 770; 20 Pac., 364.

There is nothing in the Colorado Constitution which requires that each school of medicine named in the statute passed pursuant to its provisions shall be represented by equal numbers on the State board of medical examiners; and a certificate issued by a board having upon it more representatives of one class of practitioners than of another, will protect its holder. *Brown v. People*, 11 Colo., 109.

The fact that the mode of appointment of members of a State board of medical examiners may be irregular or unconstitutional, will not warrant the prosecution of a person practising medicine under the protection of a certificate issued by such board, as the members of such board are officers *de facto*, and their acts must be treated as valid upon a collateral attack. *Id.*

MALPRACTICE.

In an action against a physician, based on his lack of care or skill, the burden of proof to show such lack is on the plaintiff. *State, Janney v. Housekeeper*, (Md.) 2 L. R. A., 587; 19 Md. L. J., 917; 16 Atl., 382.

The party who allows a surgical operation to be performed is presumed to have employed the surgeon for that purpose, and the burden of proof to show lack of consent is on the party alleging it. *Id.*

If physicians attending a woman deem it necessary, for the preservation and prolongation of her life, to perform an operation, they are justified in doing so if she consents, whether her husband consents or not. *Id.*

The degree of care and skill required of physicians is that reasonable degree of care and skill which physicians ordinarily exercise in the treatment of their patients. *Id.*

---

Cocaine Tablets.—These tablets are now largely used by careful physicians for extemporaneous preparation of any desired strength cocaine solution. The rapid deterioration of cocaine solutions make these tablets a necessity. To make a two per cent. solution of cocaine: In one fluid-drachm of water dissolve one cocaine tablet 1⅛ grain. To make a four per cent. solution of cocaine: In one fluid-drachm of water dissolve one cocaine tablet 2¼ grains. To make a ten per cent. solution of cocaine: In one fluid-drachm of water dissolve five cocaine tablets 1⅛ grain; or dissolve two 2¼ grain tablets and one 1⅛ grain tablets in one fluid-drachm of water. Parke, Davis & Co. guarantee the purity and anesthetic efficiency of their cocaine product, and will send samples of their cocaine tablets to physicians if desired.

